# *In Vivo* Spinal Posture during Upright and Reclined Sitting in an Office Chair

**DOI:** 10.1155/2013/916045

**Published:** 2013-09-24

**Authors:** Roland Zemp, William R. Taylor, Silvio Lorenzetti

**Affiliations:** Institute for Biomechanics, ETH Zurich, Wolfgang-Pauli Straße 10, 8093 Zurich, Switzerland

## Abstract

Increasing numbers of people spend the majority of their working lives seated in an office chair. Musculoskeletal disorders, in particular low back pain, resulting from prolonged static sitting are ubiquitous, but regularly changing sitting position throughout the day is thought to reduce back problems. Nearly all currently available office chairs offer the possibility to alter the backrest reclination angles, but the influence of changing seating positions on the spinal column remains unknown. In an attempt to better understand the potential to adjust or correct spine posture using adjustable seating, five healthy subjects were analysed in an upright and reclined sitting position conducted in an open, upright MRI scanner. The shape of the spine, as described using the vertebral bodies' coordinates, wedge angles, and curvature angles, showed high inter-subject variability between the two seating positions. The mean lumbar, thoracic, and cervical curvature angles were 29 ± 15°, −29 ± 4°, and 13 ± 8° for the upright and 33 ± 12°, −31 ± 7°, and 7 ± 7° for the reclined sitting positions. Thus, a wide range of seating adaptation is possible through modification of chair posture, and dynamic seating options may therefore provide a key feature in reducing or even preventing back pain caused by prolonged static sitting.

## 1. Introduction

Today, more than 75% of all employees in industrial countries have jobs that require working in a sitting position [1]. Prolonged static sitting increases the risk of musculoskeletal disorders in the neck, shoulders, arms, and legs [2, 3] but is also a known aggravating factor for subjects with pain in the lower back [4–6]. Low back pain (LBP) is prevalent in western civilizations, with most people subject to pain and/or restricted mobility of the spine at one time or another [7]. Indeed, approximately one-third of the population are known to suffer from LBP in the course of any given month [8]. Ergonomists generally suggest that spinal health can be preserved by regular movement and varying the seating posture [9], but it remains unknown whether reclining the chair's backrest can support this process. It could therefore be important for individuals to use the different sitting positions offered by office chairs to allow the spine sufficient freedom to move and change the distribution of internal loading conditions. As a result, it is plausible that seats should make changing the sitting position as easy as possible [10]. Almost every current conventional office chair offers the option to work with different backrest reclination angles [11]. However, it remains unknown whether modification of seat tilt actually alters the posture of the lumbar, thoracic, and cervical spine, and whether it can aid in spinal unloading. Thus, the aim of this study was to determine the change in spinal geometry *in vivo*, including the vertebral bodies' coordinates, the wedge angles, and the lumbar, thoracic, and cervical curvature angles sitting in an office chair between upright and reclined positions.

## 2. Materials and Methods

### 2.1. Subjects

**Figure 1:**
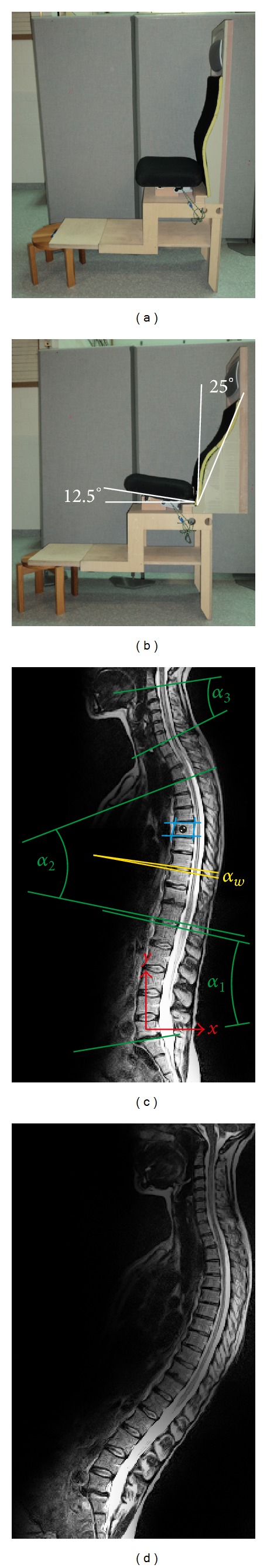
Wooden MRI-compatible prototype office chair in the upright (a) and the reclined positions (b), together with the corresponding MR images of subject 2, the only subject exhibiting vertebral degenerative changes (at the level L5-S1) (bottom), including the calculated parameters (c). Coordinate system (red arrows), lumbar (*α*_1_), thoracic (*α*_2_), and cervical (*α*_3_) curvature angles (green), exemplary wedge angle of TH9/TH10 (*α*_*w*_), and quadrangle constructed using the two endplates and the ventral and dorsal margins of the vertebral body to calculate the midpoint of each vertebra (blue quadrangle).

Five asymptomatic subjects (two females and three males) with an average age of 34 years (range 25–46 years), an average height of 1.74 m (range 1.60–1.86 m), and an average weight of 73 kg (range 55–96 kg) were analysed in an upright and reclined sitting position. Subjects with musculoskeletal disorders of the upper body, especially subjects with scoliosis as well as subjects with ferromagnetic implants, were excluded from the study. While subject 2 exhibited a degenerated L5-S1 disc (Figure 1), no subject tested within this study had any pain or functional limitation. All subjects provided written informed consent prior to participation in this study, which was approved by the ethics commission of the ETH Zurich (no. EK 2011-N-37).

### 2.2. MRI Measurements

A full-size wooden model of a new prototype office chair (Vitra AG, Switzerland; Figure 1) was constructed and used for seating analysis in a 0.6-Tesla open, upright MRI (Fonar, USA) in the Upright MRI Center, Zurich. The prototype office chair had a synchronous ratio (ratio between the backrest angle change and the seat pan angle change) of 2 and a maximal backrest reclination angle of 25° (Figure 1(b)). Eight sagittal MRI images of the lumbar, thoracic, and cervical spine were taken for each subject, who was required to sit symmetrically in each of the upright and the reclined sitting positions of the prototype chair. The T2-weighted images were recorded with a repetition time of 2530 ms, an echo time of 110 ms, and a layer thickness of 4.5 mm. The pixel spacing was 1.5 × 1.5 mm in an image plane of 360 × 360 mm. The MR images were ranked without any gaps around the median plane of the spine. The order of the measurements was performed in a randomised manner. The measurement duration for one position and spinal segment was around three minutes, in which the subjects, as far as possible, were required to maintain a static sitting position, with full backrest contact and the head in a horizontal position facing a video screen. Subjects were requested to walk around and relax between the different MRI sequences.

### 2.3. Data Analysis

The data analysis was carried out using the techniques described by Baumgartner et al. [12]. The evaluation was performed on the MR image closest to the median plane of the body using MegaCAD 2D (Version 2011, MegaCAD-Center GmbH, Oberweningen, Switzerland). The coordinates were determined based on the centre of mass of a quadrangle constructed by the two endplates and the ventral and dorsal margins of the vertebral bodies (Figure 1(c), blue quadrangle). In order to combine the three different MR images (lumbar, thoracic, and cervical), the coordinates were calculated in the coordinate system with the origin at the 5th lumbar vertebrae (Figure 1(c), red arrows). The wedge angles of the intervertebral discs were analysed for each pair of adjacent vertebrae throughout the entire spine by means of an established clinical evaluation method [13] (Figure 1(c), *α*_*w*_). The curvature angles, *α*_1_–*α*_3_, were defined as the angles between the upper L1 endplate and the upper sacrum S1 endplate, between the upper TH4 endplate and the lower TH12 endplate, and between the lower C2 endplate and the lower C7 endplate for the lumbar, thoracic, and cervical spinal, segments, respectively (Figure 1(c)). A lordosis was defined as a positive angle (*α* > 0), while kyphosis was defined as a negative angle (*α* < 0). The accuracy of the determined angles was approximately ±1° due to the resolution of the MR images.

Spinal posture was then compared in the upright and reclined seating positions for all subjects, including a correction of the reclined angle to allow an understanding of the relative geometrical changes that occur between the vertebrae.

## 3. Results

### 3.1. Coordinates of the Midpoints of the Vertebrae

**Figure 2:**
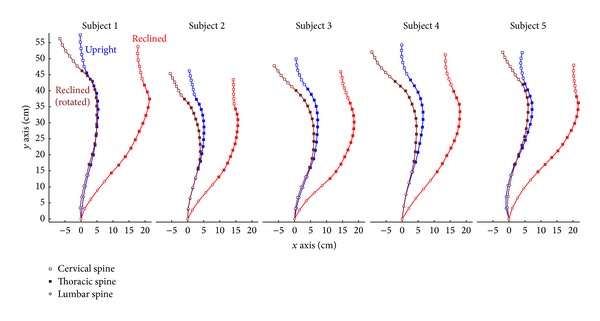
Coordinates of the midpoints of the vertebrae (L1-C2) of the upright (blue), reclined (red), together with the coordinates corrected for the 25° reclined seating positions (brown). All curves were related to the same origin, represented by the midpoint of L5.

The coordinates of the midpoints of the vertebra in the upright and reclined positions as well as the 25° rotation of the backrest in the reclined position are illustrated in Figure 2. While changing from an upright to a reclined position, the shapes of the lumbar and the lower part of the thoracic spine were very similar, as demonstrated by comparing the upright (blue lines) with the rotated reclined positions (brown lines). The coordinates of the upper (cervical) spinal segments moved forward in the reclined compared to the upright sitting position.

### 3.2. Wedge Angles

**Figure 3:**
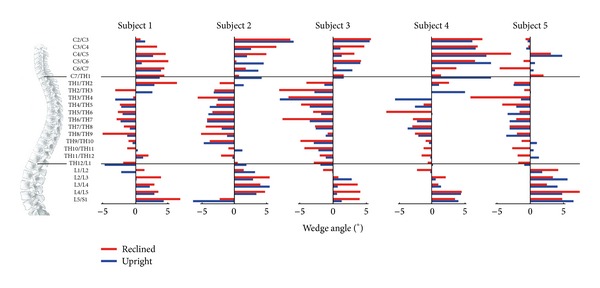
Wedge angles of the intervertebral discs shown for each subject relative to the spine locations (image adapted from [14]) for both upright (blue) and reclined (red) seating positions. Peak changes in wedge angles of up to ~12° were observed, but the locations were inconsistent across subjects.

Wedge angle changes from the upright to the reclined position occurred in every vertebral segment (Figure 3). No uniform movement pattern could be identified, as the wedge angle's variability between the subjects was very high for the whole spine.

### 3.3. Curvature Angles

**Figure 4:**
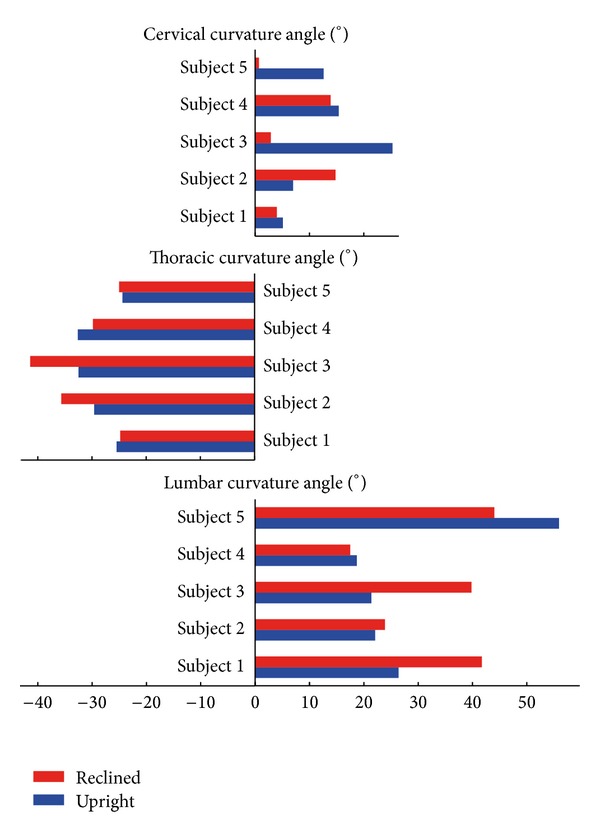
Lumbar (*α*_1_), thoracic (*α*_2_), and cervical (*α*_3_) curvature angles in the upright and reclined sitting positions for the five subjects. Note that these section curvature angles include not only the vertebral disk wedge angles, but also the geometrical curvature of the individual vertebral bodies.

The mean curvature angles for the lumbar spine, *α*_1_, were 29 ± 15° for the upright sitting position and 33 ± 12° for the reclined position (Figure 4). The changes in lumbar curvature angle from the upright to the reclined position showed a high inter-subject variability; however, two different movement strategies were detected. While subjects 2, 4, and 5 showed the same or a reduced lumbar curvature while reclining, subjects 1 and 3 increased their lumbar curvature. In the thoracic spine, the mean curvature angles, *α*_2_, were −29 ± 4° for the upright sitting position and −31 ± 7° for the reclined position. Compared to the other spinal segments, the inter-subject variability and the geometrical differences between sitting positions for the thoracic spine were very low. Conversely, the mean curvature angles for the cervical spine, *α*_3_, were 13 ± 8° for the upright sitting position and 7 ± 7° for the reclined position, suggesting high inter-subject and interposition variability.

## 4. Discussion

All intervertebral discs moved relative to one another after a change in seating posture. Similar results were demonstrated in the lower back (S1-TH11) for upright, reclined, and forward inclined sitting by Baumgartner et al. [12]. Although the two sitting positions in our set-up were clearly defined by the shape of the backrest, high inter-subject variability of the shape of the upper spinal segments was observed for both sitting positions. These findings are consistent with the high interindividual variability in the cervical spine observed by Mayoux-Benhamou et al. [15], who found a standard deviation in their cervical curvature index of more than 60% of the mean value. The forward rotation of the upper spine segments within our study in the reclined seating position is most likely a result of the headrest, which ensured that subjects maintained a horizontal head position. However, despite this slight change in headrest shape between the upright and reclined seating postures, a large variation of spine shapes was still observed between the subjects. High interindividual variability was also observed in the wedge angles throughout the spine for both sitting positions, but no specific spinal segment could be identified where the majority of the reclination movement occurred. Further investigation into understanding the cause of this variability should include a greater number of study participants but also examine the different pressure distributions on the chair to assess whether sitting on, for example, the ischial tuberosities plays a role in spinal posture.

Andersson et al. [16, 17] reported that backrest reclination and a convex lumbar support are able to increase lumbar lordosis. Their study showed a wide range of lumbar curvature changes. Our results revealed that subjects are able to make use of two movement strategies. While changes in spinal posture in the reclined seating positions seemed to result from hip joint rotation in subjects 2, 4, and 5 (same or reduced curvature while reclining), subjects 1 and 3 presented a largely lumbar segment movement (increased lumbar curvature while reclining). The thoracic curvature angles were very similar for all subjects in the two sitting positions, which could be explained by the backrest support as well as the more rigid structural anatomy of the thoracic part of the spine [18].

## 5. Conclusions

The findings of this study suggest that a wide range of seating posture adaptation is possible through modification of chair posture. While additional research is required to elucidate the role of prolonged static sitting on pathologies of the back, dynamic seating options might play a key role in maintaining spinal health, especially in subjects with desk jobs. The current study provides a basic understanding of the spinal rhythm while moving from an upright into a reclined sitting position.
